# Comparative Study of Four Time Series Methods in Forecasting Typhoid Fever Incidence in China

**DOI:** 10.1371/journal.pone.0063116

**Published:** 2013-05-01

**Authors:** Xingyu Zhang, Yuanyuan Liu, Min Yang, Tao Zhang, Alistair A. Young, Xiaosong Li

**Affiliations:** 1 Department of Medical Statistics, West China School of Public Health, Sichuan University, Chengdu, Sichuan, P.R. China; 2 Division of Psychiatry, School for Community Health Sciences, University of Nottingham, Nottingham, United Kingdom; 3 Department of Anatomy with Radiology, University of Auckland, Auckland, New Zealand; Northeastern University, United States of America

## Abstract

Accurate incidence forecasting of infectious disease is critical for early prevention and for better government strategic planning. In this paper, we present a comprehensive study of different forecasting methods based on the monthly incidence of typhoid fever. The seasonal autoregressive integrated moving average (SARIMA) model and three different models inspired by neural networks, namely, back propagation neural networks (BPNN), radial basis function neural networks (RBFNN), and Elman recurrent neural networks (ERNN) were compared. The differences as well as the advantages and disadvantages, among the SARIMA model and the neural networks were summarized and discussed. The data obtained for 2005 to 2009 and for 2010 from the Chinese Center for Disease Control and Prevention were used as modeling and forecasting samples, respectively. The performances were evaluated based on three metrics: mean absolute error (MAE), mean absolute percentage error (MAPE), and mean square error (MSE). The results showed that RBFNN obtained the smallest MAE, MAPE and MSE in both the modeling and forecasting processes. The performances of the four models ranked in descending order were: RBFNN, ERNN, BPNN and the SARIMA model.

## Introduction

Typhoid fever is a disease caused by the bacterium, *Salmonella enteric* subspecies *enteric* serovar Typhi, and is common in developing and underdeveloped countries [1]. Several typhoid fever outbreaks have been reported by the World Health Organization (WHO) over the past decades [2]. According to the WHO, an estimated 22 million cases of typhoid fever occur annually, with at least 200,000 deaths. More than 90% of these cases are estimated to occur in Asia [3]. In some underdeveloped areas of China, typhoid fever is still a serious infectious disease that severely affects lives of the patients. Stringent measures should be taken by the local government to decrease the occurrence of typhoid fever and avoid major health problems for patients based on the severity and high incidence rate of typhoid fever. Therefore, the need arises for a modeling approach that can provide decision makers early estimates of future typhoid fever incidence based on the historical time series data. The goal is to monitor and predict the trends in typhoid fever incidence to facilitate early public health responses to minimize morbidity, mortality and the adverse clinical outcomes of the patients.

Several complex statistical models have been proposed to forecast the occurrence of typhoid fever and a typical example is the type of mechanistic models proposed by Cvjetanovij [4], [5]. Those mechanistic models assume that the population is composed only of a few subgroups of key characteristics which are relevant to the infection under consideration, such as the susceptible, the infected and the immune. The models also make assumptions about relation between the subgroups, for example, the change in the number of infected during a short time interval is assumed to be proportional to the number the susceptible. To explain the course of the disease, the mechanistic models require epidemiological information about the proportions of epidemiological subgroups in the population and rates of transition between groups to decide essential transmission parameters in order to establish an explanatory model. Those assumptions may be too strong to hold for the real world situation. In China and perhaps some other developing countries, the current public health surveillance system does not collect detailed essential epidemiological information as they are often difficult to obtain. In contrast, time series forecasting is applied as an effectively non-explanatory mean to predict future epidemic behavior based on historical data. Time series are relative simple to fit and require less epidemiological information. Like many other infectious diseases, typhoid incidence time series exhibit seasonal behavior, secular trend and rapid fluctuations. Therefore it is reasonable to forecast epidemic incidence with time series methods [6]. A good forecasting performance of time series models will facilitate the understanding of the epidemic patterns by public health officials so as to make early interventions.

There are five types of traditional time series models most commonly used in epidemic time series forecasting and in other forecasting areas. They are (1) autoregressive (AR), (2) moving average (MA), (3) autoregressive moving average (ARMA), (4) autoregressive integrated moving average (ARIMA), and (5) seasonal autoregressive integrated moving average (SARIMA) models. AR models express the current value of the time series linearly in terms of its previous values and the current residual; whereas MA models express the current value of the time series linearly in terms of its current and previous residual series. ARMA models are a combination of AR and MA models, in which the current value of the time series is expressed linearly in terms of its previous values and in terms of current and previous residual series [7]. The time series defined in AR, MA, and ARMA models are stationary processes, which means that the mean of the series of any of these models and the covariance among its observations do not change with time. For non-stationary time series, transformation of the series to a stationary series has to be performed first. ARIMA model generally fits the non-stationary time series based on the ARMA model, with a differencing process which effectively transforms the non-stationary data into a stationary one. SARIMA models, which combine seasonal differencing with an ARIMA model, are used when the time series data exhibits periodic characteristics. SARIMA models have been widely used in forecasting for infectious disease and other areas when data exhibit a seasonal trend, with applications such as hemorrhagic fever with renal syndrome [8], dengue fever [9], and tuberculosis [10]. This model has been demonstrated as an effective linear model that can grasp the linear trend of the series. However, the assumption of linearity in many time series events may not be satisfied in practice. The accuracy of the statistical forecasting model therefore needs to be improved.

Models based on artificial neural networks can effectively extract nonlinear relationships in the data. They have been widely used in time series predictions because of their characteristics of robustness, fault tolerance, and adaptive learning ability [11]. Unlike the SARIMA model of linearity, neural network models have nonlinear functions that constitute the linkage between the value at time *t* and its previous value at *p* time points. The prediction model is expressed in the general form, 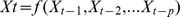, where *X_t_* represents the value at time *t* and *f* is a nonlinear function. Among artificial neural networks, the back-propagation neural networks (BPNN), radial basis neural networks (RBFNN), and Elman neural networks (ERNN) are the most commonly used methods [12]. All these three types of neural networks have successfully shown their usefulness in various types of classification and nonlinear regression problems.

BPNN use a type of backward propagation of errors and multilayered feed-forward neural networks [13]. The neural network is trained by historical data to capture the characteristics of this time series. The connection weights are adjusted iteratively by a process of minimizing the forecast errors. BPNN are widely used in economic areas [14], engineering [15], and weather forecasting areas [16]. They have also been introduced into forecasting the incident cases of Hepatitis A [17]. Many of these studies showed that the BPNN is a useful tool in time series forecasting.

RBFNN are a popular alternative approach to BPNN which use a radial basis function as its activation function [18]. RBFNN is proposed to overcome the main drawback of BPNN of easily falling into local minima in the training process. RBFNN have also been used in various forecasting areas and achieve good forecasting performance, with demonstrated advantages over BPNN in some applications [19], [20]. However, few studies have discussed the application of this type of neural network in infectious disease forecasting [21].

ERNN use a type of recurrent neural network, in which one or more additional context layers storing the delayed hidden layer values are augmented [22]. With the addition of the context layer, ERNN are more effective in learning dynamic information contained in the series. ERNN have not been applied in forecasting of infectious diseases, although they have been proven to be efficient in other forecasting applications [23].

In summary, classical traditional time-series forecasting models such as SARIMA differ from artificial neural networks time series models in both theoretical and practical aspects. Comparative studies of different forecasting techniques can facilitate the selection of the best time series model for forecasting future epidemic behavior in specific types of diseases [24]. In the present study, we address this problem by comparing the forecasting performance of the SARIMA model and three typical artificial neural networks, namely, BPNN, RBFNN and ERNN in short-term forecasting for typhoid fever, using typhoid fever incidence data for Guangxi province, China, for illustration. The theoretical and practical aspects of the four models are also explored. We found that the neural network based models outperformed the traditional SARIMA model in forecasting the typhoid fever incidence. The performances of the different types of neural networks were also different, with the RBFNN outperforming others in the study. Advantages and limitations of these models in forecasting of typhoid fever incidences are discussed.

## Materials and Methods

### Materials

We gathered available monthly incidence data of typhoid fever from the Chinese Center for Disease Prevention and Control (CDC). Guangxi province was chosen as the study area because it has one of the highest typhoid fever records in China. Guangxi is located in southwest China (20° 54'-26° 24N, 104°26′-112°04' E), occupying an area of 236,700 km^2^ with a population of over 45 million people in 2010. The typical year-round climate is subtropical rainy, which consists of long, hot summers and short winters. The annual mean temperature and rainfall are 16°C to 23°C and 1080 mm to 2760 mm, respectively.

**Figure 1:**
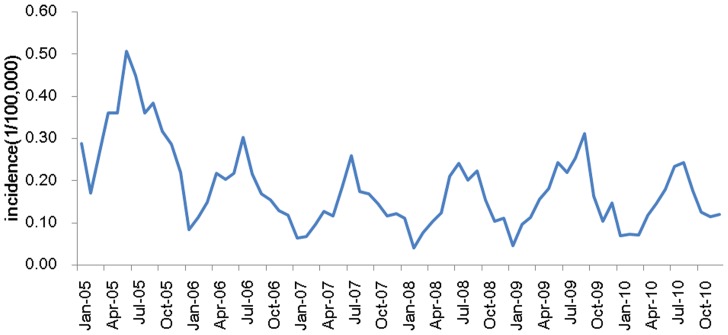
Monthly typhoid fever incidence series of Guangxi province in China from 2005 to 2010.

The data were collected from the Chinese National Surveillance System (CNSS) established in 2004 [25]. The time series data for typhoid fever incidence in Guangxi showed a strong seasonality trend, with higher incidence rates from April to September (see Figure 1). The mean annual incidence of typhoid fever in Guangxi Province was 2.17 cases per 100,000 inhabitants over a six-year period from 2005–2010. In 2009, 977 cases of typhoid fever were reported in Guangxi. The rate of reported typhoid fever in Guangxi was 1.66 cases per 100,000 people in 2010, 3.07 in 2005, 2.06 in 2006, 1.63 in 2007, 1.69 in 2008, and 2.02 in 2009. The incidence dataset between 2005 and 2009 was used as the training sample to fit the model, and the dataset in 2010 was used as the testing sample.

### SARIMA Model

The SARIMA model was developed from AR, MA, and the combination of AR and MA, the ARMA models [7]. In the AR model, the current incidence of the time series *x_t_* is a linear function of its previous incidence (*x_t−1_*, *x_t−2_*…) and the current incidence residual 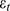. The model can be expressed as [26]:
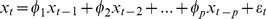
(1)


In the MA model, the current incidence of the time series *x_t_* is a linear function of both its current and previous incidence residuals 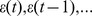. The model can be expressed as:
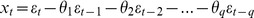
(2)


The ARMA model combines AR and MA models, in which the current incidence of the time series *x_t_* is a linear function of its previous incidence (*x_t−1_*, *x_t−2_*…) and current and previous incidence residuals 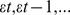 [26]. The model can be expressed as:
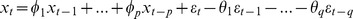
(3)


The ARIMA model deals with non-stationary time series with a differencing process based on the ARMA model.

As an extension of the ARIMA method, the SARIMA model not only captures regular difference, autoregressive, and moving average components as the ARIMA model does but also handles seasonal behavior of the time series. In the SARIMA model, both seasonal and regular differences are performed to achieve stationarity prior to the fit of the ARMA model.

The SARIMA model is usually termed as SARIMA (*p, d, q*) × (*P, D, Q*)*_S_*. In the expression, *P* is the seasonal order of the autoregressive part, *p* the non-seasonal order of the autoregressive part, *Q* the seasonal order the moving average part, *q* the non-seasonal order of the moving average part, *d* the order of regular differencing and *D* the order of seasonal differencing. The subscripted letter “*s*” indicates the length of the seasonal period. For example, in a daily data time series a weekly cycle will be expressed as *s* = 7, whereas in a monthly data time series an annual cycle be expressed as *s* = 12. In the present study, typhoid fever varies in an annual cycle, so *s* = 12. The model degenerates into an AR model when *p* is the only nonzero constant and into a moving average (MA) model when *q* is the only nonzero constant. SARIMA is applied in the present study because typhoid fever exhibits a seasonal pattern (see Figure 1).

The SARIMA modeling procedure for seasonal pattern, introduced by Box and Jenkins, consists of three iterative steps: identification, estimation, and diagnostic checking [27]. In addition, one needs to make sure that the data are stationary. This can be achieved by performing an appropriate seasonal difference in addition to the regular difference of the ARIMA model. Stationarity can be tested using Augmented Dickey-Fuller (ADF) method [28]. The identification step involves the process of determining seasonal and non-seasonal orders using the autocorrelation functions (ACF) and partial autocorrelation functions (PACF) of the transformed data [29]. The ACF is a statistical tool that measures whether earlier incidence in the series have some relation to later ones. The PACF captures the amount of correlation between the incidence at time *t* and the incidence at time *t*+*k* with the linear dependence of the incidence at time *t*+1 through to the incidence at time *t*+*k*−1 removed. After the identification step, parameters in the SARIMA model(s) are estimated using the conditional least square (CLS) method [30]. Finally, the adequacy of the established model for the series is verified by employing white noise tests [31] to check whether the residuals are independent and normally distributed. It is possible that several SARIMA models may be identified, and the selection of an optimum model is necessary. Such selection of models is usually based on the Akaike Information Criterion (AIC) and Schwartz Bayesian Criterion (SBC) [32] defined respectively as follows:
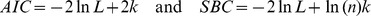
where *L* represents the likelihood function, *k* is the number of free parameters (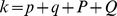) and *n* is the number of residuals that can be computed for the time series. The choice of each parameter calls for a minimization of the AIC and SBC.

The entire SARIMA modeling process can be realized using the ARIMA Procedure in SAS 9.2 [33].

### Neural Networks Based Models

Artificial neural networks were designed to mimic the characteristics of the biological neurons in the human brain and nervous system [34]. In the case of modeling the epidemic time series, the historical incidence are sent into the input neurons, and corresponding forecasting incidence is generated from the output neurons after the network is adequately trained. The network “learns” the information contained in the incidence time series by adjusting the interconnections between layers. The structure and interconnections change with the variation of the time series data in a typical data-driven and adaptive learning process. Artificial neural networks can only be viewed in terms of the input, output and transfer characteristics. The specific interconnections cannot be seen even after the training process. There is no easy way to interpret the specific meaning of the parameters and interconnections within networks trained using the real epidemic time series data. However, the advantages of neural networks for forecasting time series data are twofold: (1) they can fully extract the complex nonlinear relationships hidden in the time series, and (2) they have no need to assume the underlining distribution for the data collected [35]. The basic theories of the three types of neural networks are introduced as follows:

#### (1) Back-propagation neural networks (BPNN)

**Figure 2:**
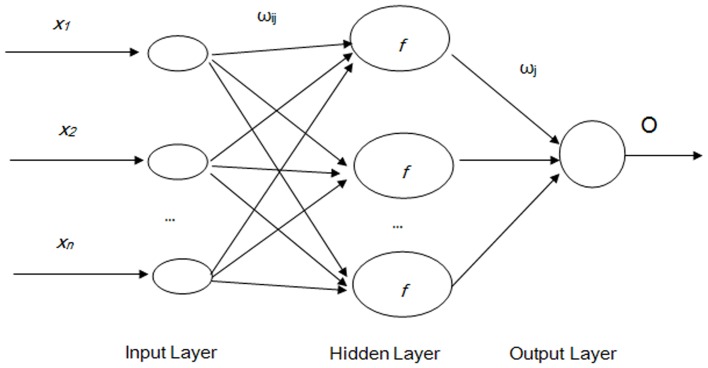
Schematic of BPNN. (Note: *x_i_* is the *i*th sample of the input layer, *ω*_ij_ is the connection weight between the *i*th input node and the *j*th node of the hidden layer, *f* is the activation function of the hidden layer, *ω*_j_ is the connection weight between the *j*th node to the output node, *O* is the output of the network.)

BPNN are a type of feed forward artificial neural networks. In feed-forward neural networks, the data flow is in one direction and the answer is obtained solely based on the current set of inputs. BPNN consist of an input layer, a hidden layer, and an output layer. Each layer is formed by a number of nodes, and each node represents a neuron. The upper- and lower-layer nodes are connected by the weights 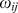 and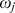, where *i = *1, 2,…, *n*, *j = *1, 2,…, *m*, *n* the number of input layer neurons, and *m* the number of hidden layer neurons. The common structure of a BPNN model is illustrated in Fig. 2 (Schematic of BPNN).

BPNN are trained with a back-propagation algorithm, in which incidences of training samples are entered from the input layer, and the outputs are calculated through the operation of corresponding functions and connection weights between the nodes. The output error is obtained by comparing the calculated values with the target outputs. The smaller the output error the better fit of the model. If the error does not meet the accuracy requirements set previously, the network weights will be adjusted along the opposite direction of the network until the required minimum network error is eventually achieved [36].

BPNN training includes three steps: (1) the forward feeding of the input training pattern, (2) the calculation and back-propagation of the associated error, and (3) the adjustment of the weights. With *n* input neurons, *m* hidden neurons, and one output neuron, the outputs of all hidden layer nodes are calculated as follows:
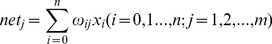
(4)

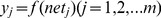
(5)where *net_j_* is the activation value of the *j*th node, 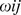 the connection weight from input node *i* to hidden node *j*, *x_i_* the *i*th input, *y_j_* the corresponding output of the *j*th node in the hidden layer, and *f* the activation function of a node, which is usually a sigmoid function



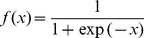
(6)The outputs of all output layer neurons are expressed as
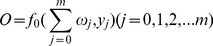
(7)where *f_0_* is the activation function, which is usually a line function; 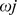 is the connection weight from the hidden node *j* to the output node, and *y_j_* is the corresponding output of the *j*th node in the hidden layer. All the connection weights are initialized randomly, and then modified according to the results of the BP training process. Several methods have been proposed for the adjustment of the connection weights, such as the steepest descent algorithm, Newton’s method, Gauss–Newton’s algorithm, and Levenberg–Marquardt algorithm [37]. These algorithms are complicated, and an introduction and comparison work can be found elsewhere [38]. In the current study, the Levenberg–Marquardt algorithm, which blends the steepest descent method and the Gauss–Newton algorithm, is selected [39]. This algorithm inherits the speed advantage of the Gauss–Newton algorithm and the stability of the steepest descent method. The Matlab software provides an effective toolbox for the realization of neural networks. BPNN with various training algorithms can be easily realized using the newff() function. The use of the Matlab neural network toolbox was introduced by Beale [40].

#### (2) Radial Basis Function Neural Networks (RBFNN)

**Figure 3:**
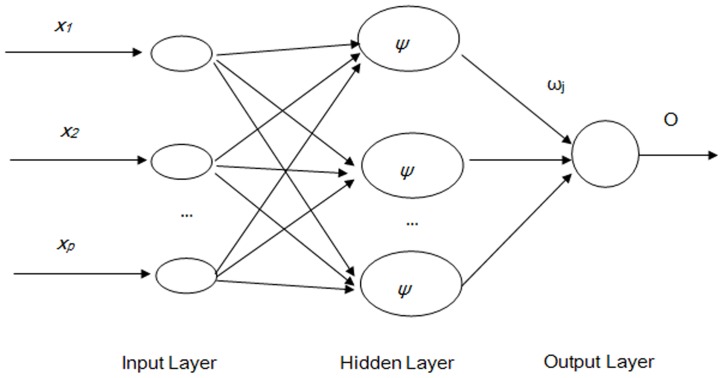
Schematic of RBFNN. (Note: *x_i_* is the *i*th sample of the input layer, *ψ* is the RBF function of the hidden layer, *ω*_j_ is the connection weight between the *j*th node to the output node, *O* is the output of the network.)

RBFNN are a special type of feed-forward neural network that uses a radial basis function as their activation function. The connections between the input and hidden layers are not weighted, and the transfer functions on the hidden layer nodes are radial basis functions in the RBFNN, which is different from those in BPNN [41]. RBFNN generally train faster than BPNN due to the use of the radial basis functions. The common structure of an RBFNN is illustrated in Fig. 3 (Schematic of RBFNN). Similar to BPNN, an RBFNN is composed of three layers: the input, hidden, and output layers. Each node in the hidden layer corresponds to a basis function, whose activation is evaluated by the distance between an input vector and the center of the basis function. The output of the network is a linear combination of the radial basis functions. Since the choice of the basis function is not very crucial to the performance of the network, the most common choice is the Gaussian function. An input vector is placed into each node of the hidden layer, and each node calculates the distance from the input vector to its own center. The resulting distance value is transformed via the Gaussian function, and output from each node. The output value from the hidden layer is multiplied by weighting values. The product is placed into the output layer node, which sums all the products [42].The output of RBFNN with the Gaussian basis functions is:
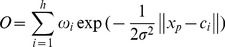
(8)where *x_p_* is the *p*th input sample of the network. *p* is the number of the radial basis functions, *c_i_* the center of the basis functions, and 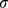 the spread of the radial basis functions. 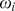 is the connection weights between the hidden and output layers.

Three main parameters in RBFNN need to be estimated in the training algorithm of the networks. They are the center of the basis function *c_i_*, the spread of the basis function 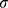, and the weights between the hidden layer and the output layer 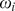. In the hidden layer, each neuron has an activation function. Different training algorithms, such as the unsupervised *k*-means cluster [43] and the supervised Orthogonal Least Squares (OLS) methods [44], have been proposed during the past decades to select the center of the basis function. The Matlab neural network toolbox includes the newrb() function to realize the RBFNN with OLS algorithm. The OLS algorithm jointly optimizes all parameters of the network, similarly to BPNN. The mathematical theory of the OLS training algorithm was introduced by Sherstinsky [45]. An appropriate spread constant must be chosen in RBFNN training. A small spread constant results in a steep radial basis curve, which forces a small number of neurons to respond to an input. A large spread constant results in a smooth radial basis curve, which allows more neurons to respond to an input. Therefore, the spread constant must be chosen to ensure that enough radial neurons respond to an input, but not so large that all of the radial neurons respond equally.

#### (3) Elman recurrent neural networks (ERNN)

**Figure 4:**
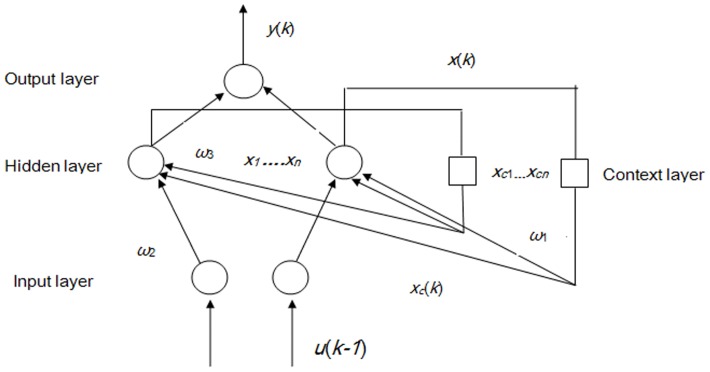
Schematic of ERNN. (Note: *u*(*k−1*) and *y*(*k*) are the input and output of the network, respectively, at a discrete time *k*; *x_c_*(*k*) and *x*(*k*) are the nodes of the context and the hidden layers, respectively; and 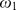,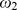, and 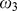 are the weight matrices for the context-hidden, input-hidden, and the hidden-output layers, respectively.)

The previous two types of neural networks are both feed forward with all input signals flowing in one direction from input to output. Feed-forward networks can perform static mapping between input and output spaces. In contrast, recurrent networks are built in such a way that the outputs of some neurons can be fed back to the same neurons or to neurons in the preceding layers, which means that signals can flow in both forward and backward directions [46]. Recurrent networks have a dynamic memory: their outputs reflect the current input, as well as previous inputs and outputs. ERNN are one of the typical recurrent neural networks that can reflect the dynamic changes in the considered systems. Compared with BPNN, ERNN have a context layer that can send the feedback from the output connections to the hidden layer [47]. The context layer functions in storing internal states in ERNN, as previously mentioned. Fig. 4 (Schematic of ERNN) illustrates the ERNN structure, where *u*(*k−1*) and *y*(*k*) are the input and output of the network, respectively, at a discrete time *k*; *x_c_*(*k*) and *x*(*k*) are the nodes of the context and the hidden layers, respectively; and 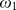, 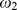, 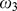 are the weight matrices for the context-hidden, input-hidden, and the hidden-output layer, respectively. The dynamics of ERNN are described by Equations (9) (10) (11):
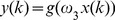
(9)

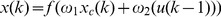
(10)

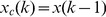
(11)where *f* is a hyperbolic tangent function. The training of ERNN is similar to BPNN, for example using the back-propagation algorithm. Different training algorithms have been developed and compared previously [48]. For the present study, we chose the Resilient Back Propagation method, which is reported as a fast and robust algorithm in previous studies [49]. The Matlab neural network toolbox includes the newernn() function to realize the ERNN with various algorithms.

### Model Selection Criterion and Evaluation Index

Different parameters or network structures can be determined each time for certain data in the SARIMA model and artificial neural networks. For the SARIMA model, the AIC and SBC, which were introduced in Section 2.2.1, act as the criteria for selecting the best model. However, in neural networks, no criteria for model selection similar to AIC and SBC exist. Usually, the modeling sample is divided into two parts: the training sample, which is used for training the sample, and the validation sample, which is used to test the efficacy of the built structure. The selection of a best structure is based on the minimization of the bias between the values obtained from the training and validation samples and their corresponding observed values from the raw data.

Furthermore, the contrast between the observed value of the raw series and the predicted values obtained through the four methods were compared to determine the efficacy of the four forecasting methods used in the present study. The mean absolute error (MAE), mean absolute percentage error (MAPE), and the mean square error (MSE) were selected as the measures of evaluation because as empirical methods they are widely used in combining and selecting forecasts for measuring bias and accuracy of models [50]. These measures were calculated using Equations (12), (13), and (14). *Pt* is the predicted value at time *t*, *Zt* is the observed value at time *t* and *T* is the number of predictions
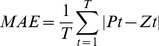
(12)

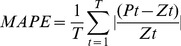
(13)

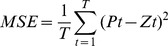
(14)


## Results

### SARIMA Model

A SARIMA model was fitted to typhoid fever incidence data from 2005 to 2009 and tested by predicting the incidence for 2010. The series after seasonal differencing were tested to be stationary (*p*<0.05) using the ADF test. Different SARIMA models were tested to determine the best fitting model. Table 1 (Estimation of available SARIMA models) presents the results of the estimations using various SARIMA processes for typhoid fever incidence. SARIMA (0, 0, 1)×(0, 1, 0)_12_ was selected as the most appropriate model and was used to forecast the monthly incidence of 2010. The parameter significance test (*t = *4.54, *p*<0.01) and the white noise diagnostic check (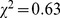, *p = *0.36) for residuals obtained by the selected model were made to ensure that the data was fully modeled.

**Table 1:** Estimation of available SARIMA models.

Model	AIC	SBC
SARIMA (1, 0,0)×(0,1,0)_12_	−144.88	−140.03
SARIMA (0, 0,1)×(0,1,0)_12_	−146.18	−144.33
SARIMA (2, 0,0)×(0,1,0)_12_	−145.62	−141.92
SARIMA (0, 0,2)×(0,1,0)_12_	−144.30	−140.60

### Development and Results of Neural Networks

Three different types of neural networks were employed to fit the incidence trend of typhoid fever. The available incidence time series was divided into three subsets. Typhoid fever incidence from January 2005 to August 2009 was employed as the training set used for training the network. The incidence from September 2009 to December, 2009 was employed as the validation set. The optimum network was determined according to the least MSE between the training and validation sets. The remaining set of the series, namely the incidence of 2010 was used as the test set.

Figs. 2, 3, 4 illustrate the structures of the adopted models. The number of inputs of the neural networks was determined by the seasonal period of the time series. The training of artificial neural networks for learning seasonality in the data structure does not require any transformation of the original incidence series [51]. In the present study, the period of the incidence of typhoid fever observed was twelve. Therefore, twelve was selected as the number of the input layer for the three types of neural networks, in which the last 12 months of data were reserved as the input for forecasting the present data. The output layer of all three types of artificial neural networks only contains one neuron representing the forecast value of the incidence of the next month. Prior to the training of neural networks, the proper transition of the data series is always necessary to determine the input and the output data. Supposing that *X_t_* represents the value at time *t*, the input matrix and the corresponding output matrix of the training and validation sample used in our study are written as follows:
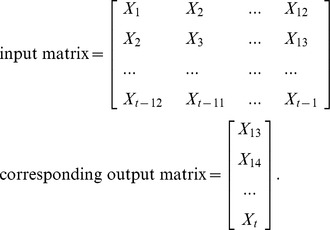



The input matrix is sent into the input layer for training, and its corresponding output matrix is its training goal. The training process starts after the input and output matrixes are placed into the corresponding Matlab neural network functions and the relevant parameters are properly set. Once the structure is determined, it is used to forecast the incidence in 2010 iteratively.

Different learning rates, learning algorithms and the number of neurons in hidden layers will affect computation efficiency in BPNN. To date, no standard rules for selecting the number of hidden neurons and layers exist. The specific network structure is generally fixed by trial and error [52]. For BPNN, different learning rate constants were examined, namely, from 0.025 to 0.5 with 0.025 increments. Different numbers of hidden layer neurons were tested in the network from 2 to 50 with an increment of 1. The BPNN 12-7-1 was selected as the optimum BPNN in the paper using the trial-and-error method. For RBFNN, different spread constants were examined in the study, namely, from 0.1 to 2.0 in 0.1 increments. The RBFNN performed best when the spread was 0.2 using trial and error. For ERNN, different learning rate constants were examined in the study, namely, from 0.025 to 0.5 at 0.025 increments. Different numbers of hidden layer neurons were tested in the network from 2 to 50 at an increment of 1. The ERNN 12-28-1 was selected as the optimum ERNN in the paper using trial-and-error.

### Comparisons of Forecasting Performance

**Figure 5:**
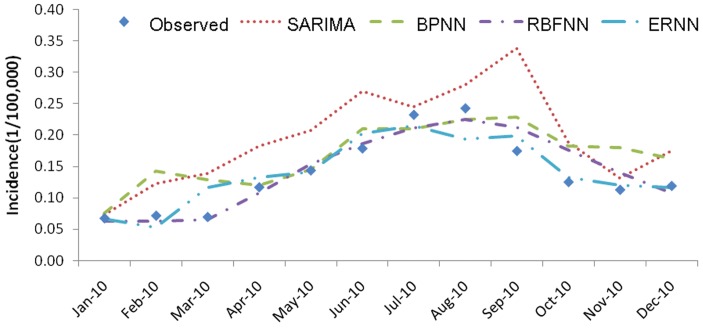
Typhoid fever incidence and fitting values for 2010 predicted by the four methods.

**Figure 6:**
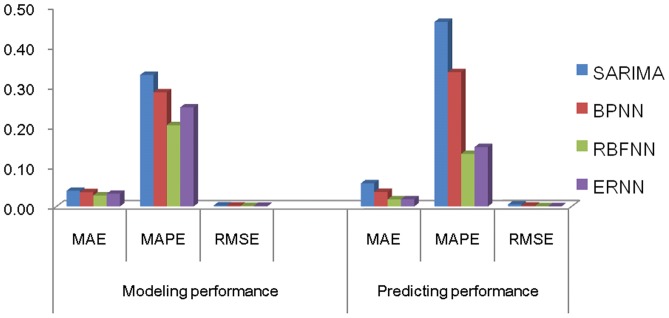
Comparison of the performances of the four different models.

**Figure 7:**
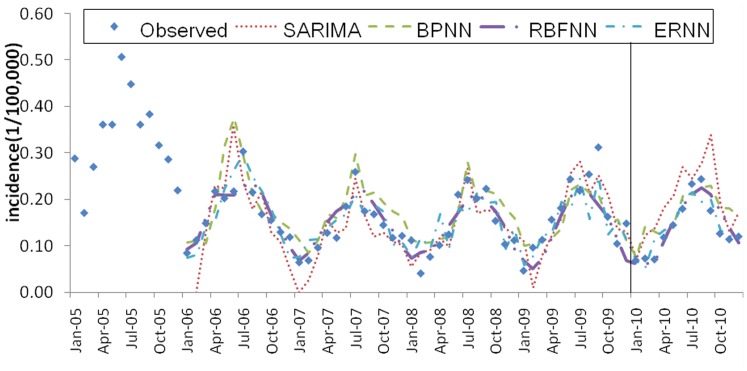
Typhoid fever incidence and fitting values predicted by the four methods. (Note: The data were divided into modeling and forecasting groups with a vertical line; the left is the modeling part, and the right is the forecasting part.)

Table 2 (Incidence values for 2010 predicted by different forecasting models) and Fig. 5 (Typhoid fever incidence and fitting values for 2010 predicted by the four methods) present the forecasting values of the four methods, as well as the observed values obtained by surveillance data. The graphs indicated that the predicted values by all the selected models reasonably matched the obtained data. Table 3 (Comparison of the performances of the four different models) and Fig. 6 (Comparison of the performances of the four different models) show the modeling and prediction performances of the four methods. It can be seen that the MAE, MAPE and MSE measures are the lowest for RBFNN among the four methods. ERNN had smaller MAE, MAPE, and MSE than SARIMA and BPNN. The SARIMA model had the largest MAE, MAPE, and MSE among the four methods. The fitting and forecasting incidences of the four methods for over six years are graphed in Fig.7 (Typhoid fever incidence and fitting values predicted by the four methods). The overall performance of the four models was ranked in descending order as follows: RBFNN, ERNN, BPNN and SARIMA.

**Table 2:** Incidence values for 2010 predicted by different forecasting models.

Time	Observed	SARIMA	BPNN	RBFN	ERNN
January	0.06796	0.07290	0.07528	0.06246	0.06694
February	0.07208	0.12273	0.14331	0.06245	0.05294
March	0.07002	0.13935	0.12874	0.06558	0.11732
April	0.11738	0.18295	0.12012	0.10819	0.13274
May	0.14415	0.20787	0.14550	0.15385	0.14224
June	0.17916	0.27016	0.21092	0.18687	0.20285
July	0.23270	0.24524	0.21039	0.21122	0.21557
August	0.24300	0.28054	0.22534	0.22541	0.19463
September	0.17504	0.33868	0.22910	0.21241	0.19882
October	0.12562	0.18918	0.18288	0.17669	0.13338
November	0.11326	0.13104	0.18043	0.13958	0.12051
December	0.11944	0.17465	0.16272	0.10800	0.11732

**Table 3:** Comparison of the performances of the four different models.

Model	Modeling performance			Predicting performance		
	MAE	MAPE	MSE	MAE	MAPE	MSE
SARIMA	0.03924	0.32906	0.00250	0.05796	0.46188	0.00498
BPNN	0.03581	0.28557	0.00224	0.03624	0.33610	0.00192
RBFNN	0.02751	0.20333	0.00127	0.01762	0.13162	0.00050
ERNN	0.03186	0.24814	0.00144	0.01790	0.14871	0.00056

## Discussion

The early recognition of epidemic behavior is significantly important for epidemic disease control and prevention. The effectiveness of statistical models in forecasting future epidemic disease incidence has been proved useful [53]. Several researchers introduced different approaches to forecasting epidemic incidence in previous studies. Exponential smoothing [54] and generalized regression [55] methods were used to forecast in-hospital infection and incidence of cryptosporidiosis respectively. Decomposition methods [56] and multilevel time series models [57] were used to forecast respiratory syncytial virus. ARIMA and SARIMA models have been widely used for epidemic time series forecasting including the hemorrhagic fever with renal syndrome [8], [58], dengue fever [9], [59], and tuberculosis [10]. Models based on artificial neural networks were also applied to forecast hepatitis A [60]. Unfortunately, few studies on typhoid fever time series forecasting have been conducted [61]. Studies focusing on the risk prediction of typhoid fever, as well as other infectious diseases, are necessary to fill up the research gap. This is especially important in areas where typhoid fever is common and brings serious social and economic burden. Moreover, as there have been many different time series models for prediction, the issue of which model will be the “best” for the prediction of epidemic incidence attracts increased attention. Where comparative studies on the accuracy of different models for forecasting epidemic behavior were carried out, inconsistence in model performance between studies has been observed. For example, SARIMA model have demonstrated better performance than generalized models in forecasting cryptosporidiosis cases in northeastern Spain [62], and better than regression and decomposition models in forecasting campylobacteriosis in the United States [63], but dynamic linear models showed better performance than the SARIMA model in forecasting hepatitis A and malaria [25]. In forecasting hepatitis A cases, the traditional multilayer neural networks emerged as the better model than the ARIMA models, radial basis neural networks and time-delayed neural networks [22]. The different findings of these studies suggest that further studies focusing on the comparison of different kinds of predicting methods for different type of diseases are necessary for the application in forecasting epidemic behavior. To bridge the research gaps, we conducted a rigorous comparative study of four time series investigations in the forecasting of the epidemic pattern of typhoid fever, namely SARIMA, BPNN, RBFNN, and ERNN, which is the first study of this kind for infectious diseases to our knowledge. We have also compared the differences among these methods in both principle and practical aspects.

In principle, the SARIMA model can grasp the historical information by (1) seasonal and regular differences to achieve stationarity, (2) AR to take into account the past values, and (3) MA to take into account the current and previous residual series. The SARIMA model is popular because of its known statistical properties and the well-known Box–Jenkins methodology in the modeling process [64]. It is one of the most effective linear models for seasonal time series forecasting. In contrast, the artificial neural network time series models capture the historical information by nonlinear functions. With flexible nonlinear function mapping capability, artificial neural networks can approximate any continuous measurable function with arbitrarily desired accuracy.

In practical matters, the building of the SARIMA model requires the determination of non-seasonal and seasonal differencing orders (*d*, *D*), and operators (*p*, *q*, *P*, *Q*), as well as the estimation of model parameters in the autoregressive and moving average polynomials. BPNN and RBFNN are both feed-forward neural networks. The construction of BPNN is based on the algorithm of back propagation, whereas RBFNN generally uses a linear transfer function and nonlinear transfer function (normally the Gaussian function) for the output and hidden layers, respectively. These characteristics provide RBFNN a number of advantages over BPNN in terms of simple architecture, learning scheme and fast training speed. ERNN also have a simple architecture. The main difference between BPNN and ERNN is the addition of the context layer in ERNN. The context units of ERNN can memorize all the feed inputs such that the outputs of the network depend upon the current input as well as the previous inputs. ERNN can learn temporal patterns effectively with memory and recurrent feedback connections. Neural networks are nonparametric nonlinear models that impose fewer prior assumptions on the underlying process from which the data are generated. Given this property, the methods in general are more tolerant to the data and less susceptible to model misspecification problems than classical time series forecasting models.

As a reflection of the concepts discussed above, the present study shows that artificial neural networks generally outperform the conventional SARIMA model in forecasting typhoid fever incidence. Although the SARIMA model has been proved an effective linear model to effectively capture a linear trend of the seasonal series, it may not work well for the occurrence of an infectious disease such as typhoid fever which can be affected by various factors, including many meteorological and various social factors, namely, the occurrence of the disease not necessarily associates with the historical data in linear relationship. Our study suggested that nonlinear relationships may exist among the monthly incidences of typhoid fever so that the SARIMA model did not efficiently extract the full relationship hidden in the historical data. Artificial neural networks have been widely accepted as potentially useful methods of modeling complex nonlinear and dynamic systems in various research fields. The good performance of artificial neural networks in the present study in forecasting typhoid fever has provided new evidence of such properties of those models.

The types of artificial neural network may significantly affect the forecasting accuracy. In the present study, three different types of models were employed to forecast the incidence of typhoid fever, and their forecasting efficacies compared based on the MAE, MAPE and MSE empirical measures. The ERNN efficiently captured the dynamic behavior of typhoid fever incidence compared with the BPNN, resulting in a more compact and natural internal representation of the temporal information contained in the incidence series. The accuracy of forecasting by ERNN could be further improved by optimizing data selection strategies and training parameters. In comparison, the RBFNN showed the best performance in term of accuracy and training time. The proposed RBFNN can also overcome several limitations of the BPNN and ERNN, such as highly non-linear weight update and slow coverage rate. Those features of RBFNN together with its natural unsupervised learning characteristics and modular network structure make it a more effective candidate for fast and robust incidence forecasting.

In conclusion, the SARIMA model has advantages in its well known statistical properties and effective modeling process. It can be easily realized through mainstream statistical software, such as SAS, SPSS, and Stata, and can be used when the seasonal time series are stationary and have no missing data. The disadvantage of the SARIMA model is that it can only extract linear relationships within the time series data. Artificial neural networks are potentially useful endemic time series forecasting methods because of their strong nonlinear mapping ability and tolerance to complexity in forecasting data. They are especially useful when a nonlinear relationship exists within the time series data. The disadvantage of the artificial neural networks is their black-box nature, in which the specific nonlinear functions within the time series data may not be explained well in practice.

The limitations of the study should also be acknowledged. First, we only obtained typhoid fever incidence data over a six-year period because the Chinese National Surveillance System for Infectious Disease was established only in 2004. The relatively short length of the series may influence the forecasting efficacy of the different methods. Second, we only predicted typhoid fever incidence with the four forecasting methods. The findings based on the specific disease may not be repeatable when used on other cases. Similar research should be conducted on more infectious diseases to ascertain performance of those models and possible factors that will impact on the model performance in practice.

Typhoid fever epidemics pose a significant threat to human health. Strategic health planning, such as vaccination costs and stocks, can be efficiently implemented with accurate estimates and help from the local government. Further research on the accurate prediction of the incidence of more infectious disease should be conducted, and more sophisticated forecasting techniques should be introduced in practice.
